# Identification of Novel Long Non-coding and Circular RNAs in Human Papillomavirus-Mediated Cervical Cancer

**DOI:** 10.3389/fmicb.2017.01720

**Published:** 2017-09-19

**Authors:** Hongbo Wang, Yingchao Zhao, Mingyue Chen, Jie Cui

**Affiliations:** ^1^Department of Obstetrics and Gynecology, Union Hospital, Tongji Medical College, Huazhong University of Science and Technology Wuhan, China; ^2^Cancer Center, Union Hospital, Tongji Medical College, Huazhong University of Science and Technology Wuhan, China; ^3^CAS Key Laboratory of Special Pathogens and Biosafety, Center for Emerging Infectious Diseases, Wuhan Institute of Virology, Chinese Academy of Sciences Wuhan, China

**Keywords:** cervical cancer, RNA-seq, lncRNAs, circRNAs, ceRNA, HPV

## Abstract

Cervical cancer is the third most common cancer worldwide and the fourth leading cause of cancer-associated mortality in women. Accumulating evidence indicates that long non-coding RNAs (lncRNAs) and circular RNAs (circRNAs) may play key roles in the carcinogenesis of different cancers; however, little is known about the mechanisms of lncRNAs and circRNAs in the progression and metastasis of cervical cancer. In this study, we explored the expression profiles of lncRNAs, circRNAs, miRNAs, and mRNAs in HPV16 (human papillomavirus genotype 16) mediated cervical squamous cell carcinoma and matched adjacent non-tumor (ATN) tissues from three patients with high-throughput RNA sequencing (RNA-seq). In total, we identified 19 lncRNAs, 99 circRNAs, 28 miRNAs, and 304 mRNAs that were commonly differentially expressed (DE) in different patients. Among the non-coding RNAs, 3 lncRNAs and 44 circRNAs are novel to our knowledge. Functional enrichment analysis showed that DE lncRNAs, miRNAs, and mRNAs were enriched in pathways crucial to cancer as well as other gene ontology (GO) terms. Furthermore, the co-expression network and function prediction suggested that all 19 DE lncRNAs could play different roles in the carcinogenesis and development of cervical cancer. The competing endogenous RNA (ceRNA) network based on DE coding and non-coding RNAs showed that each miRNA targeted a number of lncRNAs and circRNAs. The link between part of the miRNAs in the network and cervical cancer has been validated in previous studies, and these miRNAs targeted the majority of the novel non-coding RNAs, thus suggesting that these novel non-coding RNAs may be involved in cervical cancer. Taken together, our study shows that DE non-coding RNAs could be further developed as diagnostic and therapeutic biomarkers of cervical cancer. The complex ceRNA network also lays the foundation for future research of the roles of coding and non-coding RNAs in cervical cancer.

## Introduction

Cervical cancer is the third most common type of cancer and the fourth leading cause of cancer-associated mortality in women, with an estimated 526,000 new cases and 239,000 deaths in 2015 globally (Fitzmaurice et al., 2017). Cervical cancer is caused by a persistent infection with high-risk human papillomavirus (HPV), including HPV-16, HPV-18, and HPV-53. HPV16 and HPV18, the most frequently detected subtypes worldwide, contribute to 70% of all invasive cervical cancer cases (de Sanjose et al., 2010). The carcinogenesis of HPV is dependent on the activities of viral oncoproteins E5, E6, and E7 (McLaughlin-Drubin et al., 2012). However, in most cases, women infected by HPV remain asymptomatic because an adequate immune response is capable of controlling the infection, and very few cases progress to cervical cancer (Insinga et al., 2011). Previous studies have suggested that abnormal host genes as well as additional factors, such as multiparity and smoking, influence the risk of cervical cancer development (Vaccarella et al., 2006; Louie et al., 2011;de Freitas et al., 2014). Therefore, the discovery of vital diagnostic and therapeutic molecular markers could offer substantial help in controlling the infection of HPV and preventing the progression of cervical cancer.

In recent years, much attention has been paid to two new classes of RNA, long non-coding RNAs (lncRNAs) and circular RNAs (circRNAs). LncRNAs are non-protein coding transcripts that are longer than 200 nucleotides (Cao, 2014). The newly identified non-coding circRNAs are single-stranded, covalently closed circular molecules. Both can act as microRNA sponges and regulate gene expression by functioning as competing endogenous RNA (ceRNA) in distinct physiological and pathophysiological states (Li et al., 2017). Mounting evidence shows lncRNAs and circRNAs play key roles in diseases, especially in cancer, and can serve as diagnostic or predictive biomarkers (Qu et al., 2015). However, as far as we know, no research has been completed about the function of circRNAs in cervical cancer. Furthermore, the entire ceRNA network related to cervical cancer is unclear.

In this study, we performed high-throughput RNA sequencing (RNA-seq) of cervical squamous cell carcinoma (CSCC) and matched adjacent non-tumor (ATN) tissues from three patients. First, we identified differentially expressed (DE) lncRNAs, circRNAs, miRNAs, and mRNAs between CSCC and ATN tissues. More importantly, three lncRNAs and 44 circRNAs have not been reported before. Second, gene ontology (GO) and Kyoto encyclopedia of genes and genomes (KEGG) pathway enrichment analyses were performed. Additionally, co-expression and function analyses were carried out to reveal the potential biological roles of the lncRNAs. Finally, we constructed a ceRNA network of coding and non-coding RNAs to predict the interaction among lncRNAs, circRNAs, miRNAs, and mRNAs. We found that the majority of the novel non-coding RNAs may be involved in cervical cancer. Our findings revealed for the first time that circRNAs are likely to be involved in cervical cancer and DE non-coding RNAs could be further developed as biomarkers. The complex ceRNA network might help to understand the interplay among coding and non-coding RNAs in the carcinogenesis and the proliferation of cervical cancer.

## Materials and Methods

### Tissue Samples

Three pairs of freshly frozen tissues of CSCC and matched adjacent non-tumor tissue (ANT) were collected from three cervical cancer stage IB1 patients (caused by HPV16) who underwent a radical hysterectomy between June 2016 and December 2016 at Union Hospital, Tongji Medical College, Huazhong University of Science and Technology. The collected adjacent normal tissues were 2 cm away from the visible cervical cancer lesions. Once the tissues were dissected, they were immersed in RNAlater (Qiagen GmbH, Hilden, Germany) for 30 min, and they were then stored in liquid nitrogen until further use. Cervical cancer diagnosis was based on the International Federation of Gynecology and Obstetrics (FIGO) criteria. The diagnosis of all of the cases was histologically confirmed by two independent pathologists, and all of the tumor tissues were assessed by Hematoxylin and Eosin (HE) staining. The procedures were approved by the ethics committee of Union Hospital, Tongji Medical College, and informed consent was obtained from all of the patients.

### RNA Extraction and Quality Control

Total RNA was isolated using TRIzol reagent (Invitrogen, Carlsbad, CA, United States) according to the manufacturer’s protocol. The concentration and integrity of the total RNA were measured by the Qubit^®^ RNA Assay Kit in Qubit^®^ 2.0 Flurometer (Life Technologies, Foster City, CA, United States) and RNA Nano 6000 Assay Kit of the Bioanalyzer 2100 system (Agilent Technologies, Santa Clara, CA, United States).

### RNA-seq and Data Analysis

Three biological replicates of the CSCC and control samples were used for lncRNA, circRNA, and mRNA sequencing, while three biological replicates of the CSCC and control samples were mixed respectively for miRNA sequencing. Sequencing libraries of lncRNAs, circRNAs, and mRNAs were generated using the rRNA-depleted RNA by NEBNext Ultra Directional RNA Library Prep Kit for Illumina (New England Biolabs, Ipswich, MA, United States) following manufacturer’s recommendations, and the sequencing libraries of miRNA were generated using Small RNA Sample Pre Kit (Illumina, San Diego, CA, United States). RNA-seq sequencing was performed using an Illumina HiSeq 2500 (Illumina, San Diego, CA, United States). Raw data (raw reads) of the fastq format were first processed using FASTX-Toolkit (v 0.0.13)^1^. In this step, clean data (clean reads) were obtained by removing reads containing an adapter, reads containing poly-N, and low-quality reads from raw data. At the same time, Q20, Q30, and GC content of the clean data were calculated. Paired-end reads were aligned to the human genome with Tophat (v 2.0.9) (Langmead et al., 2009). Reads that were mapped to the human genome were assembled using Cufflinks (v 2.1.1) (Trapnell et al., 2010). The miRNA reads were aligned to known mature human microRNA sequences using Bowtie (v 2.0.6) (Langmead et al., 2009). Cuffdiff (v 2.1.1) was used to calculate the FPKMs (expected number of Fragments Per Kilobase of transcript sequence per Million base pairs sequenced) of both lncRNAs and protein-coding genes in each sample (Trapnell et al., 2012). Transcripts with *P*-values < 0.05 were assigned as DE. TPM normalization is used for estimating relative circRNAs and miRNAs production levels from RNA-seq data (Zhou et al., 2010). Differential expression of circRNAs was determined with DEseq2 (Love et al., 2014), and a *P*-value < 0.05 was considered to be a threshold to measure differential expression. DE miRNAs were identified using DEGseq (Wang et al., 2010), with the Q value < 0.01 and |log2(foldchange)| > 1 used as the threshold to evaluate the statistical significance of the miRNA expression differences. The whole analysis workflow was shared on Galaxy platform^2^ and available at https://usegalaxy.org/u/chmy/w/rna-seq-differential-analysis.

### Functional Enrichment Analysis

Gene ontology analysis^3^ was conducted to annotate the genes with terms under the biological process, cellular component, and molecular function categories using the DAVID bioinformatics tool (Huang da et al., 2009). KEGG enrichment analysis was also performed to predict the molecular interactions and reaction networks with KOBAS software using a hypergeometric test (Xie et al., 2011). GO terms and KEGG pathways with *P*-values < 0.05 were regarded as being significantly enriched. The -log10 (*P*-value) denotes the enrichment score representing the significance of the corresponding GO term and pathway enrichment among DE genes.

### Correlation and Co-expression Analysis

Co-expression analysis was conducted by calculating the Pearson correlation coefficient (PCC) between the lncRNAs and the known protein-coding genes according to their expression levels. The selection parameter |PCC| > 0.95 was set as the threshold in the co-expression analysis. The co-expression network was illustrated using Cytoscape (Shannon et al., 2003).

### Competing Endogenous RNA (ceRNA) Network Analysis

The potential miRNA response elements (MREs) of lncRNA, circRNAs, and mRNAs were searched using miRanda^4^. The overlapping of the same miRNA seed sequence binding site on both the lncRNA/circRNA and the mRNA was considered to be a potential lncRNA/circRNA-miRNA-mRNA interaction. The ceRNA network was illustrated using Cytoscape.

## Results

### Identification of DE lncRNAs, circRNAs, miRNAs, and mRNAs

**FIGURE 1:**
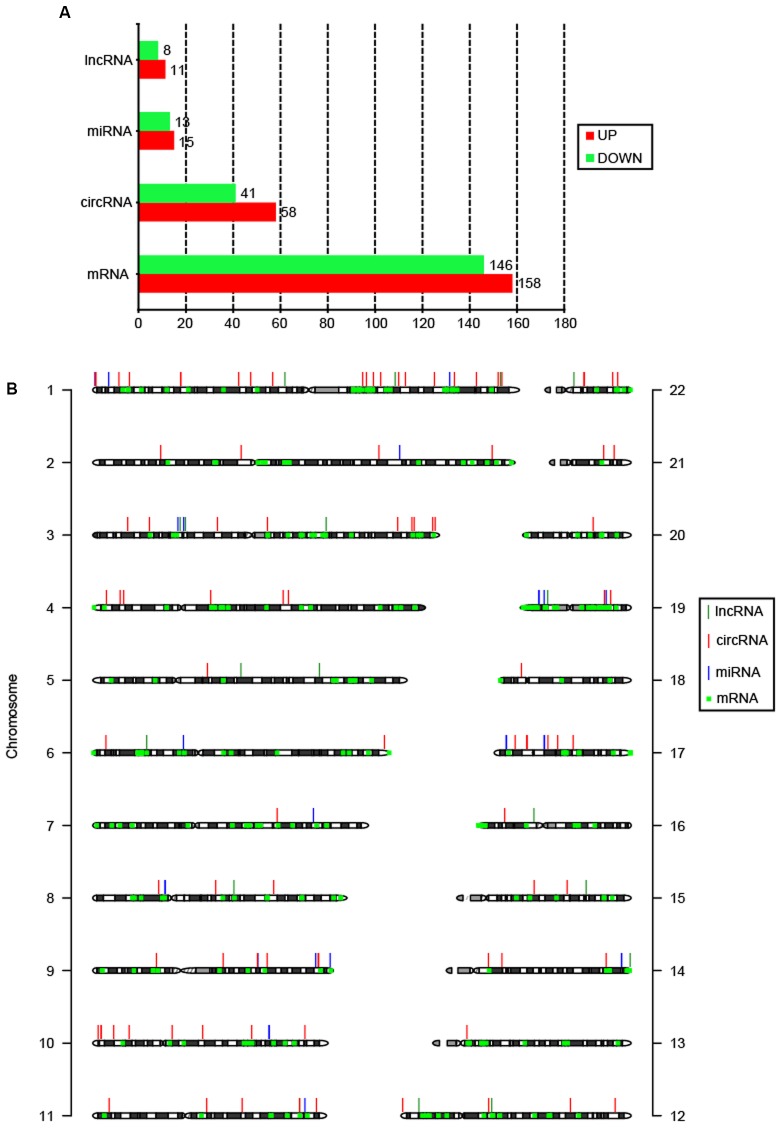
Features of DE coding and non-coding RNAs. **(A)** Number of upregulated and downregulated lncRNAs, circRNAs, miRNAs, and mRNAs. **(B)** Chromosomal distribution of DE lncRNAs, circRNAs, miRNAs, and mRNAs.

Summary of total RNA-seq data from CSCC and ATN tissues are listed in Supplementary Table S1, which demonstrated the relatively high quality of the transcriptome data. To date, there has been no report on circRNAs in cervical cancer patients. Here, for the first time, we reveal the circRNAs related to cervical cancer. RNA-seq analysis showed thousands of lncRNAs, circRNAs, miRNAs, and mRNAs expressed in cervical cancer patients. We compared the expression profiles of lncRNAs, circRNAs, miRNAs, and mRNAs between CSCC and ANT tissues from three patients, and found 19 lncRNAs, 99 circRNAs, 28 miRNAs, and 304 mRNAs were DE (**Figure 1A**). Among them, 11 lncRNAs, 58 circRNAs, 15 miRNAs, and 158 mRNAs were upregulated, and 8 lncRNAs, 41 circRNAs, 13 miRNAs, and 146 mRNAs were downregulated in the CSCC samples compared with the controls. DE non-coding RNAs are listed in Supplementary Table S2.

The DE circRNAs and mRNAs were widely scattered throughout the autosomal chromosomes, while lncRNAs and miRNAs were only distributed in 13 and 12 chromosomes, respectively (**Figure 1B**). According to their location relative to nearby protein-coding genes, lncRNAs could be classified into five categories: sense lncRNAs, antisense lncRNAs, intronic lncRNAs, intergenic lncRNAs, and bidirectional lncRNAs (Ponting et al., 2009). Among 19 DE lncRNAs, 14 lncRNAs (73.7%) were intergenic. It has been suggested that circRNAs are mainly generated from exons (exonic circRNA, ecircRNA), introns (intronic circRNA, ciRNA), or both (exon–intron circRNA, EIciRNA) (Meng et al., 2016). In this study, 64 (64.6%) DE circRNAs were EIciRNAs, and only 4 (4.0%) were ecircRNAs.

### Novel lncRNAs and circRNAs

Among the DE coding and non-coding RNAs, we uncovered 3 novel lncRNAs (LNC_000188, LNC_000226, and LNC_000231) and 44 (44.4%) novel circRNAs that have not been reported before (Supplementary Table S2). LNC_000226 and LNC_000231 were distributed in chromosome 5, and presented different trends of expression with LNC_000226 downregulated and LNC_000231 upregulated. LNC_000188 was located in chromosome 3 and upregulated. Novel circRNAs were widely scattered in 17 chromosomes. Interestingly, 25% were distributed in chromosome 1. Twenty-three (52.3%) novel circRNAs were upregulated. EIciRNAs also constitute the majority (70.5%) of the novel circRNAs.

### GO and KEGG Pathway Analyses

**FIGURE 2:**
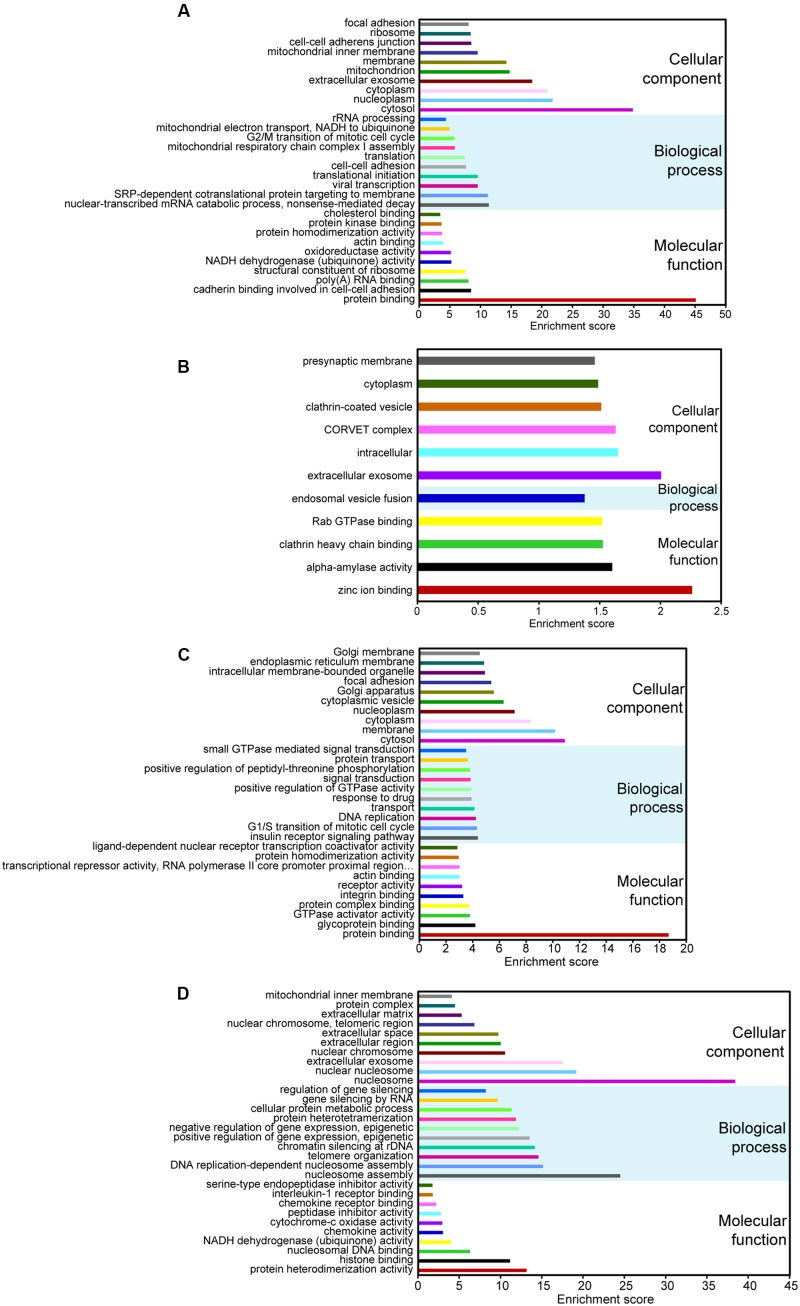
Gene ontology (GO) analyses of DE coding and non-coding RNAs. GO annotations of DE lncRNAs **(A)**, circRNAs **(B)**, miRNAs **(C)**, and mRNAs **(D)** with the top-ten enrichment score covering domains of biological process, cellular components, and molecular function. CircRNAs with less than 30 GO terms were enriched.

To predict the potential functional implication of the DE non-coding RNAs as well as mRNAs, we performed GO and KEGG functional enrichment analyses. To some extent, the function of lncRNAs could be inferred through their associated mRNAs by *cis*-regulation and *trans*-regulation (Sun et al., 2013). It is well-known that *cis*-acting lncRNAs target neighboring genes (Ponjavic et al., 2009; Orom et al., 2010); therefore, protein-coding genes 100-kb upstream and downstream of all of the DE lncRNAs were searched. We also predicted the potential targets of lncRNAs in *trans*-regulatory relationships using co-expression analysis, and the union of mRNAs resulting from *cis*-regulation and *trans*-regulation was used to conduct functional enrichment analysis. Our data showed that for DE lncRNAs, the most relevant Go terms associated with biological process included nuclear-transcribed mRNA catabolic process, SRP-dependent cotranslational protein targeting to membrane, viral transcription, translational initiation, and cell–cell adhesion (**Figure 2A**). Crucially, viral transcription and cell–cell adhesion play pivotal roles in the development and progression of cancer (Okegawa et al., 2004; Schelhorn et al., 2013). KEGG pathway analysis suggested that the most frequently predicted pathways were involved in metabolism, Huntington’s disease, and oxidative phosphorylation, Ribosome and Alzheimer’s disease, while pathways, including viral carcinogenesis and pathways in cancer, were also enriched with a relatively low enrichment score (**Supplementary Figure S1A**). These results indicate that DE lncRNAs may participate in the formation and development of cervical cancer.

Up to now, most circRNAs have not been functionally annotated (Salzman, 2016). To explore the potential function of DE circRNAs, GO and pathway analyses of circRNA hosting genes were performed. Our data suggested that only one GO term related to biological process was enriched, and it was involved in endosomal vesicle fusion (**Figure 2B**). The KEGG pathways only included nicotinate and nicotinamide metabolism and lysine degradation.

The function of DE miRNAs was also investigated. The most enriched GO terms related to biological process included insulin receptor signaling pathway, G1/S transition of mitotic cell cycle, DNA replication, transport and response to drug (**Figure 2C**). What is remarkable is that the top-two enriched pathways were proteoglycans in cancer and pathways in cancer (**Supplementary Figure S1B**). Therefore, it is also possible that DE miRNAs may be important in the pathophysiology of cervical cancer.

For DE mRNAs, strong GO enrichment focusing on biological processes was observed in nucleosome assembly, DNA replication-dependent nucleosome assembly, telomere organization, chromatin silencing at rDNA, and the positive regulation of gene expression, epigenetic (**Figure 2D**). KEGG pathway analysis showed that mRNAs were significantly enriched in the pathways involved in systemic lupus erythematosus, alcoholism, oxidative phosphorylation, non-alcoholic fatty liver disease, and viral carcinogenesis (**Supplementary Figure S1C**), which is directly correlated with tumorigenesis.

### Co-expression Analysis of lncRNAs/mRNAs and Function Prediction of lncRNAs

**FIGURE 3:**
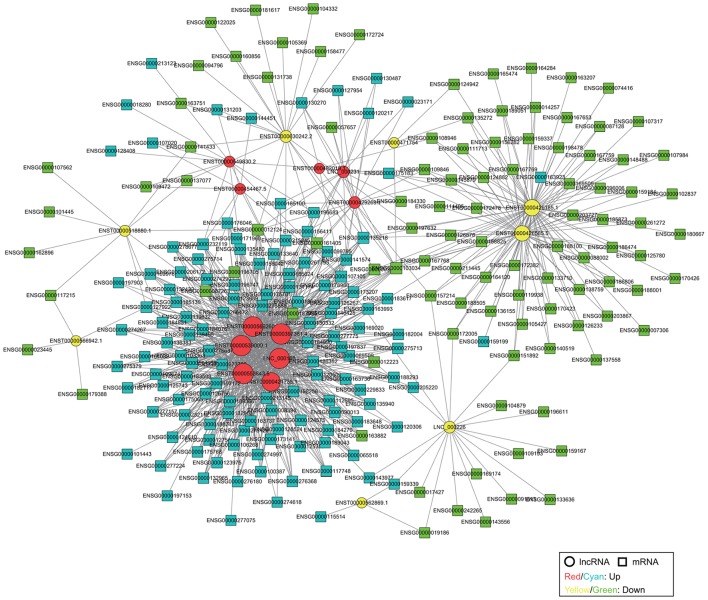
Co-expression network of DE lncRNAs and mRNAs. The network is based on Pearson correlation coefficient (|PCC| > 0.95). The size of each node is proportional to the calculated functional connectivity of each node.

We constructed a coding-non-coding gene (CNC) co-expression network based on the correlation analysis between 19 DE lncRNAs and 304 DE mRNAs. Eventually, we found 230 mRNAs (127 upregulated and 103 downregulated) that had a strong correlation with 19 lncRNAs (11 upregulated and 8 downregulated) according to the PCC analysis (**Figure 3**).

**FIGURE 4:**
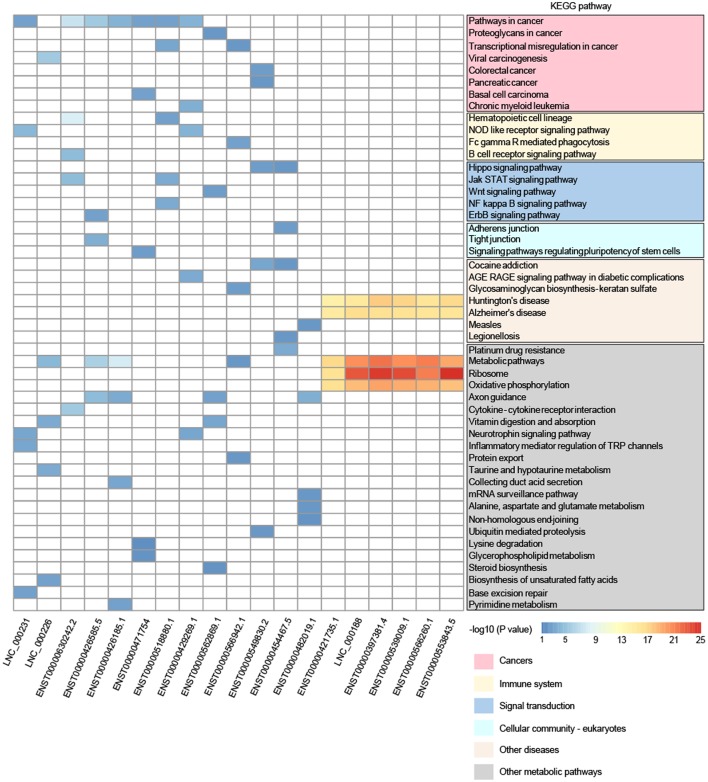
Comparison of KEGG pathway enrichment analysis of DE lncRNAs based on the target genes. Only pathways within the top-five enrichment scores are presented. The pathways could be classified into six categories: cancers, immune system, signal transduction, cellular community – eukaryotes, other diseases, and other metabolic pathways.

Furthermore, to reveal the potential function of key lncRNAs, we conducted GO and KEGG pathway analysis based on the target genes. In the KEGG pathway analysis, we only presented the top-five enriched pathways of lncRNAs, which can be classified into six categories (cancers, immune system, signal transduction, cellular community – eukaryotes, other diseases, and other metabolic pathways). It is worthy to note that 11 lncRNAs (LNC_000231, LNC_000226, ENST00000630242.2, ENST00000426585.5, ENST00000426185.1, ENST00000471754, ENST00000518880.1, ENST00000429269.1, ENST00000562869.1, ENST00000566942.1, and ENST00000549830.2) participated in at least one pathway related to cancer, and some lncRNAs may also be involved in immune system, signal transduction, and cellular community, including adherens junction (**Figure 4**), all of which may play a role in the tumorigenesis and progression of cancer. Although lncRNA ENST00000454467.5 has not been enriched in the pathway related to cancer, it was significantly enriched in the pathway involved in platinum drug resistance that is related to cervical cancer cell proliferation and apoptosis (Wang et al., 2017). The remaining seven lncRNAs (ENST00000482019.1, ENST00000421735.1, LNC_000188, ENST00000397381.4, ENST00000539009.1, ENST00000566260.1, and ENST00000553843.5) possibly interacted with mRNAs in the KEGG pathways, including Huntington’s disease, Alzheimer’s disease, ribosome, oxidative phosphorylation, and metabolic pathways, and these pathways did not seem to be implicated in cancer. However, GO analysis suggested that six lncRNAs were enriched in the GO term named viral transcription, except for lncRNA ENST00000482019.1, which may be involved in cell adhesion and signal transduction according to the GO analysis (Supplementary Table S3). Moreover, a previous study suggested that lncRNA UCA1 (ENST00000397381.4) could play an important role in the pathogenesis of cervical cancer (Wang et al., 2017). Therefore, 19 DE lncRNAs could play different roles in the tumorigenesis and development of cervical cancer, and they might even function as tumor suppressors or oncogenes. Also, the lncRNAs may represent promising potential biomarkers of cervical cancer, but further work is needed to elucidate the detailed mechanisms of the lncRNAs.

### Construction of the ceRNA Network

**FIGURE 5:**
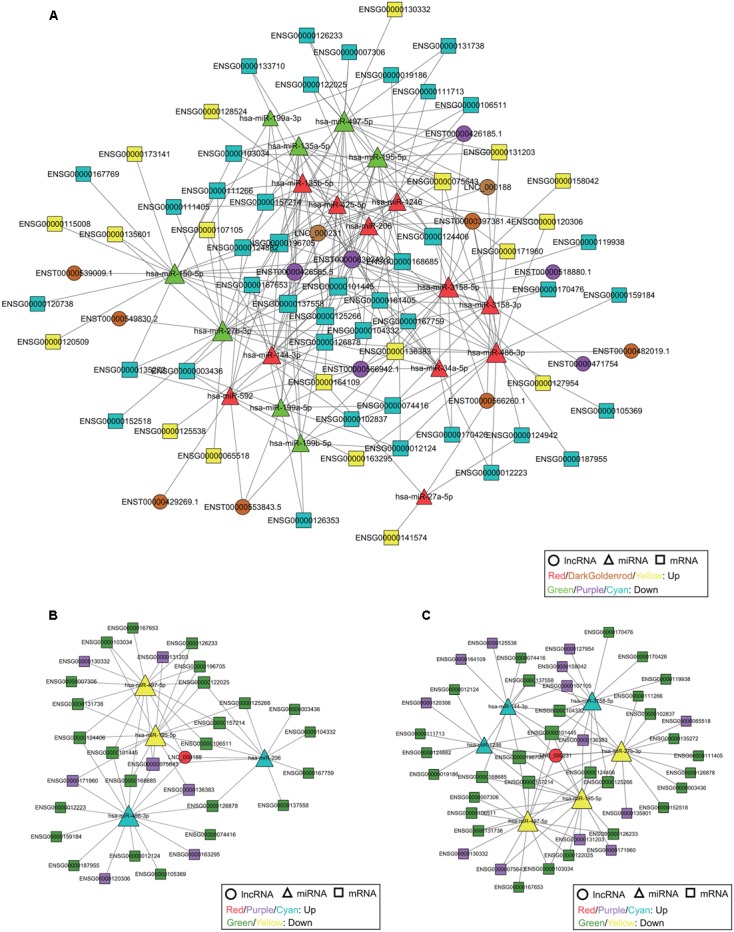
The ceRNA network, including lncRNAs, miRNAs, and mRNAs, in cervical cancer. **(A)** The ceRNA network is based on lncRNA/miRNA and miRNA/mRNA interactions. The ceRNA subnetwork of lncRNA LNC_000188 **(B)** and LNC_000231 **(C)**. The size of each node is proportional to the calculated functional connectivity of each node.

Recent studies have shown that within the ceRNA network, both lncRNAs and circRNAs can interact with miRNAs through microRNA response elements (MREs) (Salmena et al., 2011; Hansen et al., 2013). We constructed three comprehensive ceRNA networks that integrated the expression profiles and regulatory relationships of DE lncRNAs, circRNAs, miRNAs, and mRNAs from our RNA-seq data. We detected 19 miRNA-mediated lncRNA-mRNA competing triplets among 19 miRNAs, 15 lncRNAs, and 60 mRNAs (**Figure 5A**); 27 miRNA-mediated circRNA-mRNA competing triplets among 27 miRNAs, 80 circRNAs, and 62 mRNAs (**Supplementary Figure S2**); and 19 miRNA-mediated lncRNA-circRNA-mRNA competing quaternities among 19 miRNAs, 15 lncRNAs, 74 circRNAs, and 61 mRNAs (**Supplementary Figure S3**). Several of the miRNAs have been linked to cervical cancer, especially hsa-miR-1246, hsa-miR-486-3p, hsa-miR-425-5p, hsa-miR-206, hsa-miR-135b-5p, hsa-miR-150-5p, hsa-miR-195-5p, and hsa-miR-497-5p (Luo et al., 2013; Chen et al., 2014; Li et al., 2015; Ling et al., 2015; Xu et al., 2015; Ye et al., 2016; Shen et al., 2017; Sun et al., 2017), all of which have been implicated in the carcinogenesis and development of cervical cancer. Two novel lncRNAs and 28 novel circRNAs were targeted by these miRNAs, and they accounted for the majority of the novel identified non-coding RNAs, thus implying their potential function in cervical cancer. For example, hsa-miR-425-5p is upregulated in cervical cancer tissue and suggested as a potential prognostic biomarker for cervical cancer (Sun et al., 2017), but the underlying molecular mechanisms are still unclear. The potential interaction was revealed through the ceRNA network, showing that hsa-miR-425-5p targeted lncRNA HCG22 (ENST00000426185.1) and 15 circRNAs, including 5 novel circRNAs (hg38_circ_0008995, hg38_circ_0010744, hg38_circ_0011797, hg38_circ_0014951, and hg38_circ_0021570), thus enhancing our understanding of the interactions within lncRNA, circRNA, miRNA, and mRNA, especially novel lncRNA and circRNA.

LncRNA UCA1 (ENST00000397381.4) is the star biomarker in various types of cancers, such as breast cancer, gastric cancer, and lung cancer (Lee et al., 2016; Nie et al., 2016; Shang et al., 2016). Moreover, it has been proven that UCA1 is associated with cisplatin resistance, and it can be used as a potential target for a novel therapeutic strategy for cervical cancer (Wang et al., 2017). In this study, UCA1 was upregulated and interacted with hsa-miR-206, hsa-miR-3158-3p, hsa-miR-3158-5p, hsa-miR-486-3p, hsa-miR-135a-5p and hsa-miR-135b-5p (**Supplementary Figure S3**). These miRNAs also target a number of lncRNAs, cirRNAs, and mRNAs, including novel identified non-coding RNAs, thus providing potential evidence to reveal the correlation with tumor progression. Two novel lncRNAs (LNC_000188 and LNC_000231) were also included in the ceRNA network (Figure 5 and **Supplementary Figure S3**). The ceRNA network presented that lncRNAs LNC_000188 and LNC_000231 interacted with 4 (hsa-miR-195-5p, hsa-miR-486-3p, hsa-miR-206, and hsa-miR-497-5p) and 6 (hsa-miR-144-3p, hsa-miR-1246, hsa-miR-497-5p, hsa-miR-195-5p, hsa-miR-27b-3p, and hsa-miR-3158-5p) miRNAs, respectively (**Figures 5B,C**). All of the miRNAs targeted by LNC_000188 and 3 of the miRNAs targeted by LNC_000231 were involved in the carcinogenesis and proliferation of cervical cancer (Luo et al., 2013; Chen et al., 2014; Ling et al., 2015; Ye et al., 2016; Shen et al., 2017); this may explain the potential function related to cervical cancer of the two novel lncRNAs.

## Discussion

Emerging evidence shows that both lncRNAs and circRNAs play important roles in diseases, particularly cancer (Hauptman and Glavac, 2013; Wang et al., 2016; Dong et al., 2017). Recent advancements in surveying lncRNAs and circRNAs revealed that both can be used as potential diagnostic or predictive biomarkers for diseases, particularly for cancer development, progression, and prognosis. However, a comprehensive analysis of DE profiles of lncRNAs and circRNAs in cervical cancer has not been reported until now. To explore the functions and complex interactions of non-coding RNAs, we characterized the extensive transcription landscape related to the genesis and progression of cervical cancer.

We finally identified 19 lncRNAs, 99 circRNAs, 28 miRNAs, and 304 mRNAs that were DE between CSCC and ATN tissues using RNA-seq. To our knowledge, this is the first report of circRNA related to cervical cancer. Both lncRNAs and miRNAs were located in about half chromosomes whereas circRNAs and mRNAs were found throughout the autosomal chromosomes. The majority of the lncRNAs were intergenic, and most circRNAs were EIciRNAs. We also identified 3 novel lncRNAs and 44 novel circRNAs.

In order to explore the potential functions of the DE transcripts, bioinformatics methods, including GO and KEGG pathway analysis, were carried out in the study. GO analysis of lncRNAs showed that under the biological process some terms, including viral transcription and cell–cell adhesion, were enriched. Moreover, KEGG pathway analysis suggested that the pathways in cancer were also enriched. Likewise, KEGG pathway analysis of miRNAs and mRNAs revealed several pathways that could have important roles in the tumorigenesis mechanisms of cancer. This indicated that DE lncRNAs, miRNAs, and mRNAs could be correlated with cervical cancer. What was unexpected was that GO and KEGG analyses of the circRNA hosting genes only found a few enriched GO terms and pathways, all of which did not seem to be associated with cancer. One of the possibilities is that the hosting genes may be not suitable to analyze the function of circRNAs. Thus, we also used the ceRNA network to infer the biological role of circRNAs in this study.

In particular, to further reveal the function of lncRNAs, we constructed a co-expression network based on the correlation analysis between DE lncRNAs and mRNAs. There were 230 mRNAs (127 upregulated and 103 downregulated) and 19 lncRNAs (11 upregulated and 8 downregulated) that were presented in the co-expression network, and several of them have been found to be involved in cancer in previous studies. For example, Dickkopf1 (DKK-1, ENSG00000107984) acts as a new biomarker in human breast cancer (Liu J.T. et al., 2017), and it also exhibits transcriptional repression by epigenetic inactivation in specific cervical cancer cell lines, thus it may contribute to the constitutive activation of the Wnt signaling pathway in cervical carcinogenesis (Lee et al., 2008). A large number of studies have demonstrated that chemokines are involved in the progression, migration, and survival of cancers, such as CXCL12 (ENSG00000107562), CCL21 (ENSG00000137077), and especially CCL19 (ENSG00000172724), which is associated with progression of cervical cancer (Teicher and Fricker, 2010; Xiong et al., 2017; Zhang et al., 2017). Homeobox B13 (HOXB13, ENSG00000159184) is a susceptibility gene for prostate cancer (Xu et al., 2013). The co-expression network showed the interaction between lncRNAs and mRNAs, for example, CCL21 interacted with lncRNA KIAA0125 (ENST00000630242.2) and RP11-363E6.3 (ENST00000518880.1), and HOXB13 interacted with lncRNA HCG22 (ENST00000426185.1) and TPTEP1 (ENST00000426585.5). Thus, a co-expression network provides a better understanding of the biological functions of lncRNAs and mRNAs in cervical cancer. GO and KEGG pathway analyses suggested that 19 DE lncRNAs could play different roles in cancer development. Therefore, the lncRNAs may represent promising potential biomarkers for the diagnosis, treatment, and prognosis of cervical cancer, but further work is needed to elucidate the detailed mechanisms of lncRNAs.

Until now, only several ceRNAs related to cervical cancer have been revealed (Liu Q. et al., 2017; Shen et al., 2017; Yang et al., 2017), and the complex ceRNA network related to cervical cancer remained unclear. For the first time we systematically reported the ceRNA network based on DE lncRNAs, circRNAs, miRNAs, and mRNAs. Part of these miRNAs have been linked to cervical cancer, and they all target a number of lncRNAs, circRNAs, and mRNAs, including the majority of the novel identified non-coding RNAs; this presents the functional complexity of mRNAs and non-coding RNAs, and also indicates that these novel lncRNAs and circRNAs may be involved in cervical cancer and worthy of further investigation. There are some limitations in our study. Some elaborate experiments, such as overexpressing lncRNAs, need to be performed to elucidate the molecular mechanisms underlying the roles of non-coding RNAs. There is the possibility that some of the differences in RNA expression found in this study might not reflect the exact differences between normal cells and cancer cells because the cancer tissues contained not only cancer cells but also surrounding mesenchymal cells as well as inflammatory cells. Our future work will focus on further validating the miRNA-mediated ceRNA network and exploring the role of this network in the progression and metastasis of cervical cancer.

## Conclusion

This study presents a profile of DE coding and non-coding RNAs, which include 3 novel lncRNAs and 44 novel circRNAs. A series of integrated analysis found that DE transcripts, including the majority of the novel identified non-coding RNAs, may be associated with the tumorigenesis and development of cervical cancer. DE non-coding RNAs could be further developed as prospective biomarkers. This study broadens our understanding of non-coding RNAs participating in the occurrence, development, and prognosis of cervical cancer, and it will pave the way to develop novel clinical diagnostics and therapeutic approaches.

## Availability of Data

The raw reads from RNA-seq are available in the National Center for Biotechnology Information (NCBI) Sequence Read Archive (SRA) (accession number: SRP114925). The authors declare that all other data are available in the article and its Supplementary Materials or from the corresponding author on reasonable request.

## Ethics Statement

This study was carried out in accordance with the recommendations of “ethics committee of Union Hospital” with written informed consent from all subjects. All subjects gave written informed consent in accordance with the Declaration of Helsinki. The protocol was approved by the “Union Hospital, Tongji Medical College.”

## Author Contributions

HW, YZ, and JC conceived and designed the experiments. HW, YZ, MC, and JC performed the experiments. MC and JC analyzed the data. YZ and JC contributed reagents/materials/analysis tools. MC and JC wrote the paper.

## Conflict of Interest Statement

The authors declare that the research was conducted in the absence of any commercial or financial relationships that could be construed as a potential conflict of interest.

## Footnotes

1 http://hannonlab.cshl.edu/fastx_toolkit/index.html

2 http://www.usegalaxy.org

3 http://www.geneontology.org

4 http://www.microrna.org/microrna/home.do

## Supplementary Material

FIGURE S1 KEGG pathway analyses of DE coding and non-coding RNAs. KEGG pathway enrichment analysis of DE lncRNAs **(A)**, miRNAs **(B)**, and mRNAs **(C)** with a top-ten enrichment score. MiRNAs with less than 10 pathways were enriched.

FIGURE S2 The ceRNA network, including circRNAs, miRNAs, and mRNAs, in cervical cancer. The ceRNA network is based on circRNA/miRNA and miRNA/mRNA interactions. The size of each node is proportional to the calculated functional connectivity of each node.

FIGURE S3 The ceRNA network, including lncRNAs, circRNAs, miRNAs, and mRNAs, in cervical cancer. The ceRNA network is based on lncRNA/miRNA, circRNA/miRNA, and miRNA/mRNA interactions. The size of each node is proportional to the calculated functional connectivity of each node.
