# Supplementary figures and images for: Identification of Novel Long Non-coding and Circular RNAs in Human Papillomavirus-Mediated Cervical Cancer
Source: Front Microbiol. 2017 Sep 19;8:1720. doi: 10.3389/fmicb.2017.01720 (PMC5609541; doi:10.3389/fmicb.2017.01720)

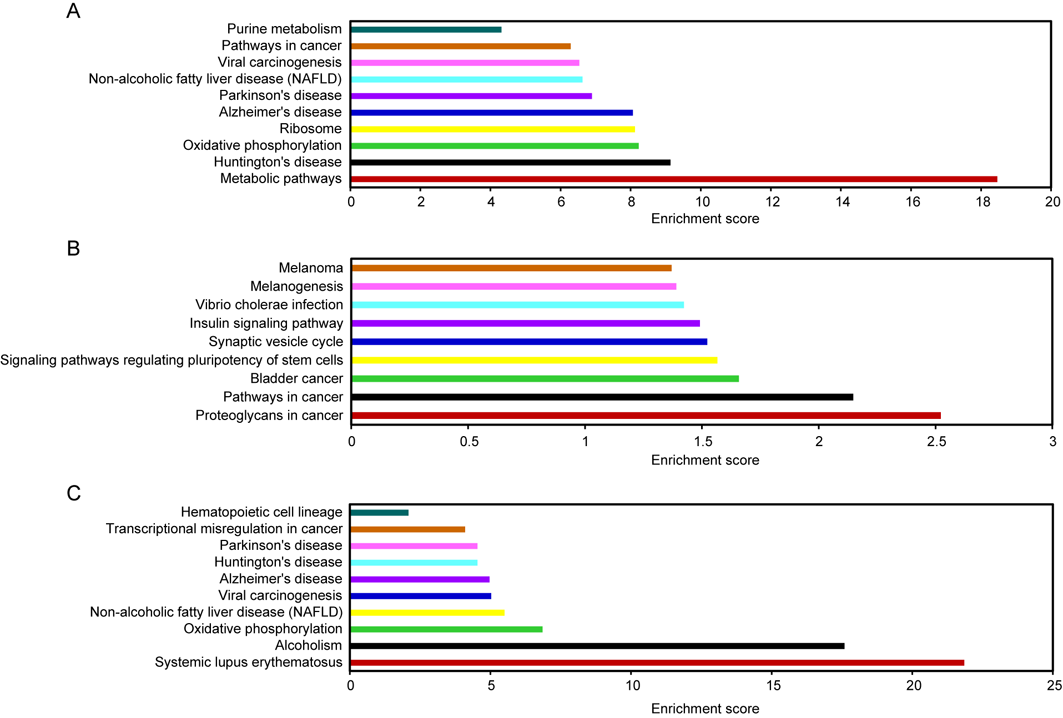

Supplement: FIGURE S1 — KEGG pathway analyses of DE coding and non-coding RNAs. KEGG pathway enrichment analysis of DE lncRNAs (A), miRNAs (B), and mRNAs (C) with a top-ten enrichment score. MiRNAs with less than 10 pathways were enriched. [file Image_1.TIF]
