# Supplementary material for: Identification of Novel Long Non-coding and Circular RNAs in Human Papillomavirus-Mediated Cervical Cancer
Source: Front Microbiol. 2017 Sep 19;8:1720. doi: 10.3389/fmicb.2017.01720 (PMC5609541; doi:10.3389/fmicb.2017.01720)
Supplement: Supplementary file 4 [file Table_1.DOCX]

**Table S1.** Summary of total RNA-seq data from CSCC and ATN tissues.

| Sample^a^ | Raw reads | Clean reads | Number (%) of Mapped reads | Q20 (%) | Q30 (%) | GC content (%) |
| --- | --- | --- | --- | --- | --- | --- |
| PT6 | 111,990,818 | 108,436,228 | 98,527,604 (90.86) | 97.19 | 93.03 | 46.64 |
| PT7_2 | 95,428,860 | 91,834,872 | 82,603,058 (89.95) | 97.23 | 92.94 | 49.54 |
| PT9 | 103,455,190 | 99,255,174 | 89,987,571 (90.66) | 96.96 | 92.37 | 53.92 |
| PN6 | 111,083,968 | 106,940,316 | 97,547,880 (91.22) | 97.18 | 92.86 | 46.71 |
| PN7_2 | 88,026,038 | 85,275,038 | 78,165,592 (91.66) | 97.30 | 93.07 | 48.03 |
| PN9 | 113,406,442 | 109,172,936 | 98,876,820 (90.57) | 97.22 | 92.94 | 49.29 |
| P_T | 13,525,229 | 13,298,698 | 11,993,514 (90.19) | 98.28 | 96.83 | 50.00 |
| P_N | 15,624,625 | 15,349,234 | 13,528,133 (88.14) | 98.29 | 96.77 | 50.44 |

^a^, Samples PN6, PN7_2 and PN9 were isolated from CSCC tissues of three patients, respectively while samples PT6, PT7_2 and PT9 from ATN tissues of three patients. Sample P_T were from mixed sample of PT6, PT7_2 and PT9 while sample P_N from mixed sample of PN6, PN7_2 and PN9.
